# Emotional scene remembering: A combination of disturbing and facilitating effects of emotion?

**DOI:** 10.3389/fnbeh.2022.992242

**Published:** 2022-10-06

**Authors:** David Bouvarel, Jeremy Gardette, Manon Saint-Macary, Pascal Hot

**Affiliations:** ^1^LPNC, CNRS, Université Grenoble Alpes, University of Savoie Mont Blanc-Chambery, Grenoble, France; ^2^Institut Universitaire de France, Paris, France

**Keywords:** attentional capture, visual complexity, trade-off effect, diffuse emotion, focal emotion

## Abstract

An emotion-induced memory trade-off effect is frequently reported when participants have to memorize complex items that include both neutral and emotional features. This bias corresponds to better remembering of central emotional information accompanied by poor performance related to neutral background information. Although the trade-off effect has been mainly associated with attentional bias toward emotional content, findings suggest that other non-attentional cognitive processes could also be involved. The aim of this work was to assess whether emotional effects would be reported apart from their influence on attentional processing in an immediate delay memory task. Three studies were conducted. In Study 1, manipulation of the diffusion quality of emotional content allowed us to select focal emotional pictures vs. diffuse emotional pictures, which prevented attentional focus. The two studies that followed consisted of a recognition task of low- and high-complexity pictures in which we used partial visual cues during the test that could display either the emotional elements (i.e., central patch cues, Study 2) or the peripheral elements (i.e., peripheral patch cues, Study 3) of the focal emotional pictures. Results from Studies 2 and 3 replicated traditional trade-off effects only for high-complexity pictures. In addition, diffuse emotional pictures were associated with lower memory performance than were neutral pictures, suggesting that emotion features could both disturb and enhance (via their attentional effect) encoding processes.

## Introduction

Cumulative evidence shows that emotional content does not improve the encoding of whole information. Rather, when competitive neutral and emotional information are present in items to be memorized, an emotion-induced memory trade-off has been repeatedly reported (Kensinger et al., 2007; Waring and Kensinger, 2009; Payne, 2011; Mickley Steinmetz and Kensinger, 2013; Cunningham et al., 2014): More resources are allocated to the emotional information at the expense of the neutral information (Waring and Kensinger, 2009, 2011). Trade-off effects in memory were first considered as an attentional bias in favor of central emotional elements. For instance, research on the *weapon focus effect* pointed out that visual focus was directed to the emotional content (i.e., the weapon), whereas peripheral details such as the individual holding the weapon were less explored (Saunders, 2009; Kocab and Sporer, 2016; Mansour et al., 2019; for reviews, see Fawcett et al., 2013). Furthermore, emotional information had more contrast than neutral information did, leading to a pop-out effect: Salient emotional information captures attention (i.e., *attentional magnets*, Schmidt and Saari, 2007; Talmi et al., 2007; Hamann, 2009; Mennie, 2015; see also *attentional narrowing*, Winograd, 1981; Chipchase and Chapman, 2013). Research further demonstrated that emotional content kept attention, leading to fewer resources being allocated to peripheral or neutral processing (e.g., see *attentional blink*, Schwabe et al., 2011; Kennedy et al., 2020; or emotion-induced blindness, Wang et al., 2012). Furthermore, attentional bias toward emotion was also evidenced by using eye-tracking technique (ET). It shows that eye-gaze is directed toward emotional content rather than toward other neutral content (Everaert and Koster, 2015; Sears et al., 2019; Skaramagkas et al., 2021; see also review conducted with affective disorders patients, Armstrong and Olatunji, 2012). In particular, increased probability of first fixation, increased fixation times (Nummenmaa et al., 2006), as well as deviations in saccade trajectory (McSorley and van Reekum, 2013), for emotional stimuli compared to neutral stimuli reflect an overt attentional bias toward emotional information (see Skaramagkas et al., 2021 for review). These results were confirmed by Isaacowitz and colleagues: first fixation and following visual exploration are oriented toward unpleasant pictures even when they are presented with competitive pleasant pictures (Isaacowitz et al., 2006, 2017; Isaacowitz, 2012, for a review, see Gurera and Isaacowitz, 2019). In addition, Chipchase and Chapman (2013) found memory trade-offs for negative stimuli concomitantly with increased number of fixations and gaze durations at the time of encoding for these stimuli. These data suggest that the distribution of overt attention during encoding is decisive in predicting memory trade-off with negative emotion.

Some authors, however, suggest that the allocation of attentional resources is not the only factor underlying emotion-induced memory trade-offs. By encouraging relevant elaboration about peripheral content presented alongside the emotional elements, Mickley Steinmetz and Kensinger (2013) showed that post-stimulus elaboration could diminish trade-off effects in memory (see also An et al., 2020). Similarly, Steinberger et al. (2011) reported that giving cognitive reappraisal strategies to participants faced with arousal content reduced subsequent memory trade-off effects. Even the seminal work of Loftus et al. (1987) on weapon focus effects raised doubt about the exclusive role of attention. In 2011, Riggs et al. (2011) conducted a mediation analysis on the role of attentional focalization in trade-off effects. The results highlighted that these attentional processes alone could not explain emotional enhancement in memory (Riggs et al., 2011). This assumption is corroborated by studies that strictly controlled overt attentional resources, which reported that recall for negative events is superior to that for neutral events, even with equal fixation time (Christianson et al., 1991; Mickley Steinmetz et al., 2014).

However, there is compelling evidence that complex emotional processing can be performed outside overt attentional mechanisms (e.g., Bisley, 2011; Rigoulot et al., 2012; D’Hondt et al., 2016; Rai and Callet, 2018; Smith and Rossit, 2018). The influence of covert attention is inefficiently controlled in traditional memory trade-off paradigms. Moreover, Talmi (2013) argued that even non-attentional effects reported in these paradigms could be the consequence of attentional biases preceding them. It was thus proposed that immediate-delay tests could not reveal non-attentional cognitive factors involved in trade-off. This hypothesis is challenged by trade-off effects reported in specific high arousal conditions for short delays between encoding and recall (Christianson et al., 1991; Mickley Steinmetz et al., 2014).

To unravel the difference between specific emotional effects and effects due to attentional capture, we built an immediate memory recognition task in which attentional effects were controlled in three different ways by using: (1) “diffuse emotion” pictures, (2) a recognition patch paradigm, and (3) stimuli with various levels of visual information complexity. First, a validation study (Study 1) allowed us to build two kinds of emotional pictures: those associated with focal emotion and those associated with diffuse emotion. A *focal emotion* picture is defined as a picture in which the emotion is concentrated in a specific and limited area (e.g., a weapon), whereas in *diffuse emotion* pictures, emotion arises from multiple sources in the picture. In the focal condition, an emotion-induced memory trade-off should be observed, whereas the diffuse condition is constructed to control attention without orienting visual exploration to a specific area of the picture. Second, we used a recognition memory task with partial visual cues (i.e., circular patches extracted from studied pictures or new patches). Using partial visual cues allowed us to measure emotional effects on recognition memory without again presenting whole pictures, which could have led to new encoding and hence interfere with recognition. This moreover allowed us to precisely isolate trade-off effects, that is, enhancement in central content and deterioration in peripheral content. In Study 2, with the focal condition, we aimed to assess whether emotional central patches effectively led to memory enhancement. At the same time, a similar enhancement measured in the diffuse condition would have supported those effects being produced without specific attentional allocation. In Study 3, with the focal condition, we aimed to assess whether neutral peripheral patches effectively led to a memory decrease when the competitive emotional area could be focused. However, in the diffuse-emotion condition, because the whole pictures contained emotion, we expected the same enhancement as in Study 2. In addition, Study 3 was based on the same material as in Study 2, thus ensuring greater reliability of the initial results. We predicted that the focal condition in Study 2 would lead to better recognition performance than the diffuse condition would, whereas both conditions would lead to better recognition than the neutral condition would. In Study 3, we expected poorer recognition in the focal condition than in the diffuse and neutral conditions and better recognition in the diffuse condition than in the neutral condition.

Finally, since emotional and attentional processes interact, there are preferential selection effects of information by the attentional system when the information is emotional (Schupp et al., 2006; Flaisch et al., 2009; Vuilleumier and Huang, 2009; Carretié, 2014; Domínguez-Borràs et al., 2020; Turkileri et al., 2021). We assumed that these effects are only present when the attentional system is forced to sort and select relevant information due to resource saturation. We then proposed two complexity conditions (low and high) and assumed that trade-off effects would be exacerbated in the high-complexity condition compared with the low-complexity condition.

Throughout these three studies, our paradigm manipulated six conditions: *diffuse*-*high*, *focal*-*high*, *neutral*-*high*, *diffuse*-*low*, *focal*-*low*, and *neutral*-*low*. We expected these studies, together, to confirm that emotion induces a trade-off that promotes emotional memories compared with neutral memories, as well as to confirm that emotion promotes retention in diffuse conditions beyond attentional biases. Moreover, we expected these studies to allow us to explore selective processing guided by emotional stimuli and to confirm that emotional prioritization of attention is strengthened in the exploration of complex stimuli.

## Study 1: Validation of focal and diffuse emotion stimuli

### Objective

In order to investigate the effect of emotion on attentional capture, it was crucial for us to ensure that our pictures would prompt focal or diffuse emotion with similar arousal and valence ratings. In Studies 2 and 3, we hypothesized that adding a diffuse emotion condition would allow us to reveal non-attentional effects in memory trade-off. Therefore, the aim of this validation study (Study 1) was to identify, among a large set of pictures, two kinds of emotional pictures: focal emotional, in which a specific emotional area elicits emotion, and diffuse emotional, in which the multiplicity of negative elements contained in the whole picture elicits emotion, but has no specific source of emotion that could capture attention. The three studies were done in line with the Declaration of Helsinki and were approved by the local ethics committee (CER_2021_15).

### Methods

#### Participants

To reduce spurious effects from a protocol duration that was too long, we chose to assess the initial set of 180 pictures in five separate sets, with 30 different participants evaluated in each set. Because there was an insufficient number of pictures that met our inclusion criteria, we performed a second session with 84 more pictures divided into two groups of 30 participants. The total number of participants in pretest sessions was 227 (mean age = 25.19 years, *SD* = 9.10, 85.2% women). University students were given credit for their participation. All participants gave their written informed consent to participate in the study after detailed information was provided to them.

#### Stimuli

**FIGURE 1:**
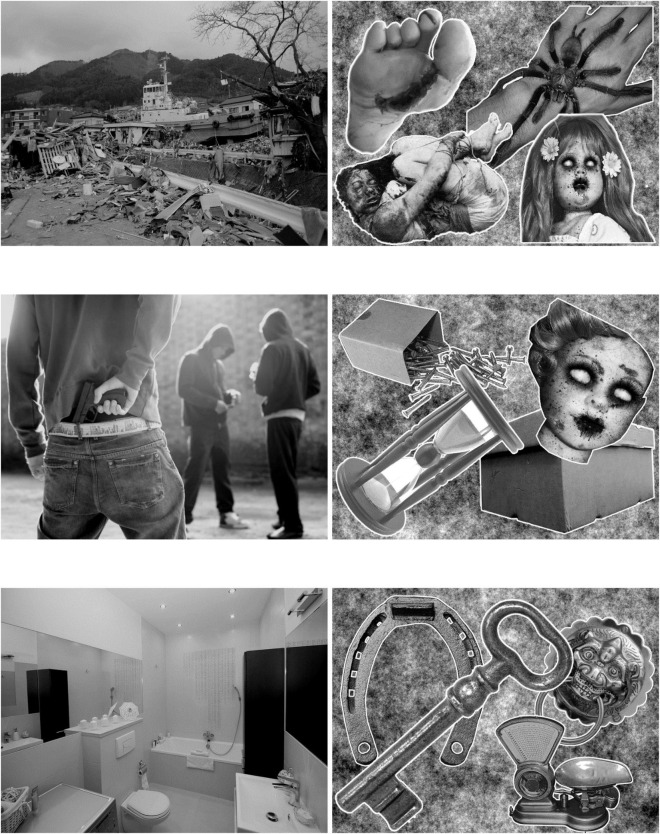
From left to right and top to bottom, diffuse-high, diffuse-low, focal-high, focal-low, neutral-high, and neutral-low. Each picture was assessed by Study 1.

We tested 264 items divided into three emotional conditions (focal, diffuse, and neutral) and two levels of complexity (low level and high level), so that six conditions were constructed: *diffuse-high, focal-high*, and *neutral-high*, and *diffuse-low, focal-low*, and *neutral-low* (Figure 1). Pictures were selected from different copyright-free databases on the internet, and 21 pictures were added from validated databases (3 from the Geneva Affective Picture Database, (Dan-Glauser and Scherer, 2011); 18 from the International Affective Picture System, Lang et al., 2008). Material is fully available by following this OSF link: https://osf.io/2rh9m/.

#### Procedure

**FIGURE 2:**
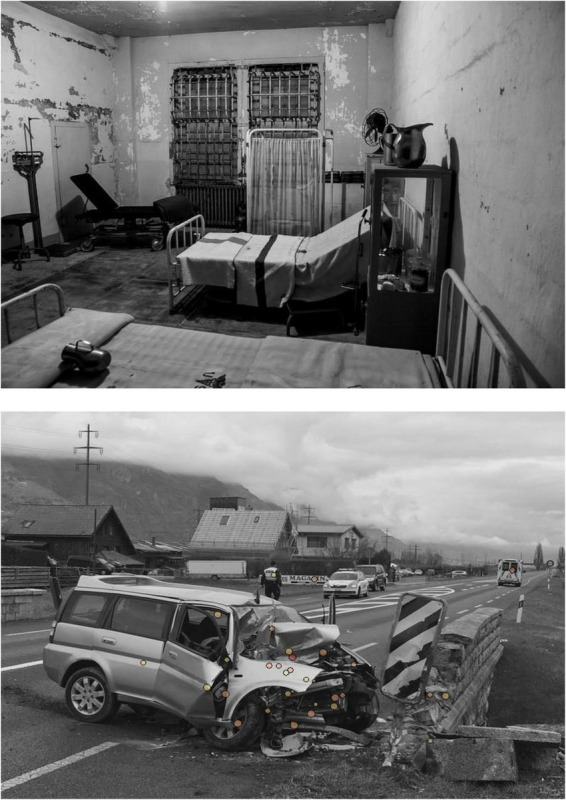
**Upper panel:** Example of a picture judged by participants to contain diffuse emotion. No specific area is highlighted, showing that participants considered the emotion to be spread through diverse, not precise, elements. **Lower panel:** Example of a picture judged by participants to be focal emotion. The source of the emotion is revealed by the sum of the points attributed on each image by participants. Different colors of the marks correspond to various levels of valence, from −4 (red) to 4 (green). No one rated this picture as positive, and so the color ranges from −4 (red) to −2 (yellow). Mark size revealed the arousal level, from 1 (the smallest points) to 8 (the largest points).

An online survey was conducted on Qualtrics.^1^ Each item was presented for 4 s followed by a maximum of five questions to answer, concerning: (1a) whether the picture contained emotion, (1b) its valence, and (1c) its arousal; (2a) for emotional pictures: whether the participant felt that the emotion was focused in a localized region of spread in the picture; and (2b) for focal pictures: where the source of emotion was (Figure 2). Participants localized the source of emotion by placing marks on each picture. Picture never disappears during this assessment. Note that these questions were skipped if participants judged the picture to be neutral. For valence, we used a Likert-like scale ranging from −4 (very negative) to + 4 (very positive), 0 being neutral (i.e., no emotional pictures). No opinion could be expressed by participants in the neutral answers from −1 to 1, and so we considered only those pictures rated from −4 to −2 on the valence scale as emotional (Raaijmakers, 2000; Kulas et al., 2008; Chyung et al., 2017). For arousal, we used a scale ranging from 1 (very calm) to 8 (very aroused) in which participants judged their feeling about the picture.

#### Results

For each picture, we calculated a frequency of emotion (i.e., number of participants indicating that they felt emotion divided by the total number of participants), as well as a focal/diffuse ratio (i.e., number of focal/diffuse responses among all responses for each emotional picture). We first identified 113 pictures considered by our sample to be emotional (mean = 80.85%, SD = 8.54%). Among these pictures, we then selected those with the highest diffuse ratio for our set of diffuse emotional pictures [i.e., above 65%: diffuse-high (*n* = 17, mean = 85.81%, SD = 8.07%); diffuse-low (*n* = 17, mean = 75.40%, SD = 3.90%)] and those with the highest focal ratio for our set of focal emotional pictures [i.e., above 65%: focal-high (*n* = 17, mean = 73.68%, SD = 8.73%); focal-low (*n* = 17, mean = 86.86%, SD = 8.01%)]. Due to heterogeneity between the effects of different emotional states (Cunningham et al., 2013), we chose to focus on pictures associated with withdrawal behavior, that is fear-like pictures. To control for arousal, we selected pictures with moderated arousal (between 3 and 6). Similarly, we controlled for valence, by selecting pictures with a valence score between −4 and –2. These criteria led us to retain 17 pictures per condition (Figure 1).

We also processed pictures to highlight sources of emotion based on responses collected during the pretest sessions. Through this process, marks were added to each picture where participants considered the picture to contain emotion and then pointed to the specific location. This analysis confirmed that diffuse-emotion conditions did not have specific emotion marks (Figure 2, upper panel); that is, participants considered emotion to be elicited by the overall atmosphere from the different elements and thus to emanate from the picture as a whole rather than from a recognizable specific object that could capture their attention (e.g., a weapon, a face, or a frightening animal). In contrast, focal-emotion conditions contained specific marks (Figure 2, lower panel) that allowed us to precisely isolate emotional areas. As a result, we were able to crop patches from this region related to the emotional area (Study 2) or not (Study 3) for our focal condition. In contrast, patches from diffuse conditions in both studies contained emotional elements. In addition, participants confirmed that pictures of the diffuse conditions did not contain restricted, localized sources of emotion that captured their attention.

## Study 2: Central patch-cued recognition task

### Methods

#### Participants

A power analysis was conducted on the basis of the work by An et al. (2020), revealing that 44 participants were needed to achieve > 90% statistical power (G*power, α = 0.05). Consequently, we recruited 79 participants (mean age = 31.36, SD = 6.85, 62% women) on Prolific^2^ so that the numbers would be more flexible in case of technical issues. The participants were paid 5£ each. An auto-reported question controlled whether they looked away during the encoding phase. To avoid incomplete processing of pictures, we excluded 28 participants because they looked away. Thus, analyses were performed on 51 participants.

#### Stimuli

**FIGURE 3:**
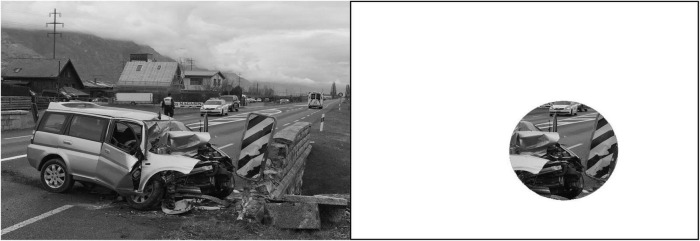
**Left:** Focal-high pictures that served as a source of patch construction. **Right:** The patch extracted from pretested pictures and its size in relation to the source picture size. Patches were constructed to give enough cues to allow recognition without providing complete pictures that could lead to new encoding.

From the 17 pictures per condition, we cropped circular patches (with a diameter of 250 pixels; see Ross et al., 2018, for a comparable method) by using Photoshop.^3^ The patches consisted of fragments (Figure 3) cut from pictures to use as partial cues for recognition but without allowing emotional processing of the entire picture. Notably, the information contained in the patches had to give enough cues for the picture to be possibly recognizable. The recognition task involved *old* patches extracted from previous preselected pictures and *new* patches constructed from non-selected but pretested pictures. Overall, 174 patches were constructed: 60% old (i.e., 102 stimuli) and 40% new (i.e., 72 stimuli).

In Study 2, patches from focal-emotion conditions were selected in the center of the emotional area. To ascertain that focal patches were emotional, we cropped them on the basis of a previous heat map obtained from Study 1. Our previous validation confirmed that diffuse-emotion conditions did not contain specific central emotional portions, but rather a combination of elements that elicited emotion. The patches extracted on the basis of those conditions naturally contained emotion.

#### Procedure

Study 2 was conducted on LABjs, a freely available tool for online experimentation.^4^ The paradigm duration was between 20 and 25 min. It was diffused online on Prolific *via* Open Lab^5^ servers. Written informed consent was collected from all participants prior to participation.

Study 2 included an encoding phase followed by a recognition phase. During the encoding phase, the 102 pictures were presented for 4 s each. Participants were instructed to watch the pictures. Each picture was preceded by a 500 ms fixation cross. A random question appeared in 20% of cases to maintain the attention of the participants. In the second section, patches were presented in a recognition memory task involving old and new patches. Finally, a few socio-demographic questions followed the completion of the two sections (i.e., social status, degree, affective pathology). The final question controlled for whether the participants looked away during the encoding phase.

#### Results

The recognition memory task was performed with patches of previous pictures. We measured the hit rate (i.e., proportion of successfully recognized old patches) and the false-alarm rate (FA; proportion of new patches incorrectly considered as old). We then calculated a discrimination index: *d’* = *HIT–FA*. We measured trade-off by comparing *d’* between the emotional and neutral conditions (as in Mickley Steinmetz et al., 2016; Gutchess et al., 2018; Madan et al., 2020). Using the lme4 package (Bates et al., 2015) in R (R Core Team, 2014), we conducted a mixed regression analysis that predicted the discrimination index as a function of *Emotion (Diffuse, Focal, Neutral)* and *Complexity (Low, High)*, with the by-subject intercept and by-subject effects of emotion and complexity as random effects.

**FIGURE 4:**
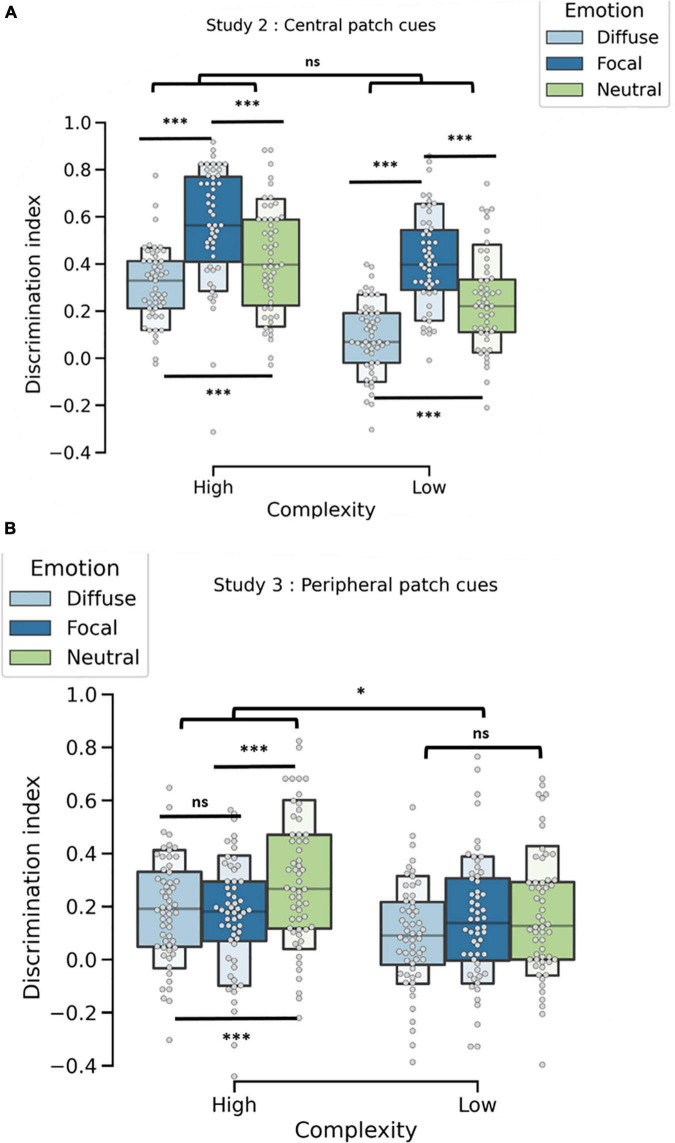
**(A)** Discrimination index (d-prime) as a function of emotional content (diffuse, focal, or neutral) for Study 2, where patch cues were centered on the emotion. Focal conditions were systematically better recognized than were mean diffuse and neutral conditions. Diffuse conditions were less well remembered than neutral conditions. High-complexity conditions were also better recognized than low-complexity conditions, but emotion and complexity did not interact. **(B)** Discrimination index (d-prime) as a function of emotional content (diffuse, focal, or neutral) for Study 3, where patch cues were not centered on the emotion. In contrast to Study 2, focal conditions in Study 3 were not better recognized than the other conditions. This difference is in line with the trade-off effect, in which peripheral information is less well encoded than central information when emotional and neutral content are in competition. Recognition was also better for high-complexity conditions than for low-complexity conditions. The interaction between emotion and complexity was held by the neutral condition. No effect = ns; *p < 0.05; ***p < 0.001.

Statistical analysis (Figure 4A) revealed a main effect of emotion, *F*(2, 49) = 56.56, *p*< 0.001. Better recognition performance was found in the focal-emotion condition (M_focal_ = 0.48, SD = 0.23) than in the other two conditions, *F*(1, 50) = 89.27, *p* < 0.001. In addition, diffuse-emotion pictures (M_diffuse_ = 0.19, SD = 0.19) were less well recognized than focal pictures, *F*(1, 50) = 121.92, *p* < 0.001. Recognition was better for neutral (M_neutral_ = 0.32, SD = 0.23) than for diffuse emotion pictures, *F*(1, 50) = 29.10, *p* < 0.001.

This analysis also revealed a main effect of complexity, *F*(1, 50) = 103.01, *p* < 0.001, stemmed by better recognition for high-complexity pictures (M_high_ = 0.43, SD = 0.23) than for low-complexity pictures (M_low_ = 0.24, SD = 0.23). There was no significant interaction between emotion and complexity, *F*(2, 100) = 1.77, *p* = 0.17. Thus, the effect of emotion on recognition performance was not modulated by the complexity of the material.

*Post hoc* analyses were conducted to compare focal vs. neutral conditions in the high and low conditions separately. In the high-complexity condition, discrimination was higher for focal emotional (M_focal–high_ = 0.56, SD = 0.24) than for neutral pictures [M_neutral–high_ = 0.41, SD = 0.23), χ^2^(1, 50) = 13.86], *p* < 0.001. The same pattern was found in the low-complexity condition for focal (M_focal–low_ = 0.41, SD = 0.20) and neutral pictures (M_neutral–low_ = 0.24, SD = 0.20), χ^2^(1, 50) = 19.21, *p* < 0.001. Thus, no trade-off differences were found between high- and low-complexity conditions.

## Study 3: Peripheral patch-cued recognition task

### Methods

#### Participants

The same power analysis was used as in Study 2, on the basis of the study by An et al. (2020): 44 participants were needed in order to achieve > 90% statistical power. Consequently, 79 participants (mean age = 26.36, SD = 5.75, 58% of women) performed Study 3 via Prolific (see text footnote 2), who received 5£ each. Twenty-three participants were excluded because they reported looking away during the encoding phase. Fifty-six participants were thus included in the final analyses.

#### Stimuli and procedure

The experimental procedure was identical to Study 2 but with a varied selection of patch locations. Whereas patches from Study 2 were chosen at the center of the emotional area in focal-emotion pictures, patches in Study 3 were located in the periphery of the emotion to replicate the reduced encoding part of the emotion-induced memory trade-off effect.

#### Results

Similar analyses as in Study 2 were performed. They revealed a main effect of emotion, *F*(2, 54) = 8.14, *p* < 0.001 (Figure 4B). Diffuse conditions led to poorer recognition (M_diffuse_ = 0.14, SD = 0.20) than neutral conditions did (M_neutral_ = 0.23, SD = 0.24), *F*(1, 55) = 15.31, *p* < 0.001. Diffuse conditions and focal conditions did not differ, *F*(1, 55) = 0.60, *p* = 0.44. Focal conditions (M_focal_ = 0.15, SD = 0.22) also led to poorer recognition than neutral conditions did, *F*(1, 55) = 10.44, *p* < 0.01.

As in Study 2, Study 3 showed a main effect of complexity, *F*(1, 55) = 12.72, *p* < 0.001, in which high-complexity conditions (M_high_ = 0.21, SD = 0.22) were better recognized than low-complexity conditions (M_low_ = 0.13, SD = 0.22).

A significant Emotion × Complexity interaction effect was found, *F*(2, 110) = 3.90, *p*< 0.05. *Post hoc* analyses revealed that the difference in high-complexity conditions for focal (M_focal–high_ = 0.16, SD = 0.21) vs. neutral conditions (M_neutral–high_ = 0.29, SD = 0.24) was greater than in the low-complexity conditions, *F*(1, 55) = 10.44, *p*< 0.05. Unlike high-complexity conditions, focal-low (M_focal–low_ = 0.15, SD = 0.23) did not differ from neutral-low conditions (M_neutral–low_ = 0.17, SD = 0.23), *F*(2, 110) = 2.99, *p* = 0.053.

## Discussion

The trade-off effect is defined as the enhancement of the emotional content of an image when it competes with neutral content. The latter is less well remembered than is emotional content (Kensinger, 2004, 2007; Waring and Kensinger, 2009, 2011; Talmi and McGarry, 2012; Chipchase and Chapman, 2013). The existence of attentional biases in favor of emotion provides a partial explanation for this trade-off (Loftus et al., 1987; Steblay, 1992; Levine and Edelstein, 2009; Saunders, 2009; Schwabe et al., 2011; Fawcett et al., 2013; Cross et al., 2018), but the literature also mentions non-attentional factors (Christianson et al., 1991; Riggs et al., 2011; Steinberger et al., 2011; Mickley Steinmetz and Kensinger, 2013; Mickley Steinmetz et al., 2014; An et al., 2020; Kensinger and Ford, 2020). The aim of our studies was to assess attentional and non-attentional effects of emotion in an immediate memory trade-off situation by controlling attentional processing with the use of “diffuse emotion” pictures during a patch-cued recognition paradigm. Additionally, we manipulated picture complexity in order to determine the conditions under which those attentional effects of emotion are optimal.

Our preliminary study (Study 1) was designed to control arousal, valence, and emotional source (i.e., diffuse vs. focal) of our pictures. This allowed us to select diffuse emotion pictures which were similar to the focal emotion pictures regarding emotional quality but without attentional capture by one specific area. Diffuse emotion conditions were constructed to assess in following studies the influence of non-attentional factors in trade-off effects by controlling attentional factors without constraining visual exploration. Considering putative attentional and emotional cumulative effects in trade-off, we assumed that diffuse conditions would reveal persistent emotional effects in absence of attentional capture by emotion.

In our two subsequent recognition tasks, we used visual patches as memory cues. These partial visual cues allowed us to manipulate the availability of the emotional element of previously encoded focal pictures during recognition with central (Study 2) vs. peripheral (Study 3) cues. In this work, we considered that an emotion-induced memory trade-off effect occurred when central information was better remembered than peripheral information in focal conditions compared with neutral conditions. Our findings confirmed that a classical trade-off effect occurred in focal conditions but that these effects were modulated by the manipulation of complexity. We assumed that in the case of high complexity, the whole picture could not be processed in the allocated time (i.e., 4 s); the attentional capture by emotion therefore led to its priority processing. The limited attentional resources led to the prioritization of the emotional area, which reinforces the trade-off. In low-complexity conditions, the smaller amount of information would allow participants to process more information within the presentation time, minimizing the impact of the attentional capture by emotion. Our findings confirm that the trade-off effect was restricted to high-complexity conditions since no difference between neutral and focal conditions were reported in the low-complexity condition. High-complexity conditions may have benefited from greater association (Luck and Vogel, 1997; Rosen et al., 2018; Robin and Olsen, 2019; Enke et al., 2020) and/or greater elaboration (Drivdahl et al., 2009; Migita et al., 2011; Nawa and Ando, 2019; Rubin et al., 2019). By revealing a modulation of the trade-off effect by picture complexity, our study suggests that such effects may be optimal in high-complexity conditions when memory is evaluated immediately. Ultimately, this observation could help to explain some discrepancies in the literature (Waring and Kensinger, 2009; Talbot, 2018).

Dealing with differences between diffuse vs. focal emotion, our finding confirmed that attentional capture is well controlled since these two conditions did not lead to similar memory performances. As expected, no trade-off was observed in diffuse conditions, whatever the level of complexity. We replicated differences between the diffuse and focal conditions in Study 3, providing strong support for the absence of a persistent emotional effect in the diffuse condition. These findings support that the main influence of emotion in short-delay memory recognition tasks relates to attentional capture.

However, our results also highlighted that performance in the diffuse conditions were systematically lower than that of the neutral conditions. Considering that emotion was present all-over diffuse pictures, we expected those to benefit from an emotional-enhancement effect (LaBar and Cabeza, 2006; Kensinger and Kark, 2018; McGaugh, 2018; Kensinger and Ford, 2020; Hall et al., 2021, for a review, Ack Baraly et al., 2017), and thus predicted the opposite result. A disturbing role of emotion has already been reported in the literature (e.g., Mather, 2009; De Lissnyder et al., 2012; see also for a review: Dolcos et al., 2020). For instance, memory for affective material can be reduced compared to neutral stimuli due to regulatory processes elicited facing emotional content, which draw resources away (Wierzba et al., 2018; Bartholomew et al., 2021; see review, Etkin et al., 2015). Memory for item-item associations can also be impaired by negative emotion (e.g., Palombo et al., 2021), which leads to an impairment in episodic memory coherence (Bisby et al., 2018). Among factors modulating the complex emotion and memory relationship, arousal is one of the most important (for a review, see Ack Baraly et al., 2017). For instance, high-level of arousal is associated with stronger consolidation, leading to better recall after a delay compared to low-level of arousal (LaBar and Cabeza, 2006; Sommer et al., 2008; Tyng et al., 2017; Turkileri et al., 2021, but for a review, see also Dolcos et al., 2017). However, an extreme level of arousal is sometimes associated to impoverished recall performance (Rauch et al., 2006; Liberzon and Sripada, 2008; Yehuda et al., 2015). In case of post-traumatic stress disorder, emotional and neutral content interfere, so that neutral stimuli incorrectly trigger emotional responses (Kleim, 2013; Hall et al., 2018; Hoffman et al., 2019). Valence is another important factor. During false memories implementation paradigm, chance of distortion and errors are bigger for negatively valenced content compared to positive one (for review, see Bookbinder and Brainerd, 2016). Conversely, research on flashbulb memories revealed that distortions are reduced when participants are affected by unpleasant event compared with pleasant one (Levine and Bluck, 2004; Kensinger and Schacter, 2006, for review of flashbulb memories, see Hirst and Phelps, 2016). In this line, our results show that both enhancing and disturbing effects could occur in a sole immediate recognition memory paradigm. We assume that the diffuse emotion condition revealed the disturbing effect of emotion, beyond its classical attentional capture.

Whereas in delayed memory tasks an enhancement effect of emotion is mainly reported (for review see McGaugh, 2018), results are less consistent in immediate memory tasks (Sharot and Yonelinas, 2008; Waring and Kensinger, 2009; Yonelinas and Ritchey, 2015). Different processes have been proposed to explain effects of emotion on immediate memory (Talmi, 2013) and long-term memory (McGaugh, 2018). The effect of emotion on delayed memory is classically explained by the effects of hormonal and amygdala activity elicited by emotional arousal on memory consolidation (for a review, see Ack Baraly et al., 2017). By contrast, the effect of emotion on immediate memory would result from specificities of affective materials such as its distinctiveness and its relevance compared to neutral materials (Talmi, 2013). Talmi and colleagues showed that the way emotion elicited these factors is strongly dependent of the type of tasks. For example, effects of distinctiveness are reduced in block designs where stimuli are gathered by emotional conditions. The disturbing effects reported in our study could thus result from the modulation of these factors rather than from consolidation effects. Further investigation, using diffuse emotion during a delayed memory task, are required to assess this hypothesis.

Our results could also be linked to the way emotional memory was tested in our studies. In particular, there is evidence showing that the enhancement of memory by emotion is reduced in recognition tasks compared to free recall tasks (Hogan and Kintsch, 1971; Carey and Lockhart, 1973; Rawson and Zamary, 2019; Rhodes et al., 2019). Thus, one hypothesis is that our immediate recognition task, by reducing enhancement effects, has allowed to unmask competitive disturbing effects. Again, additional studies would be relevant to test whether those disturbing effects apply to different memory tasks.

In sum, by strictly controlling attentional processing, we found that a competitive presentation of neutral and emotional information could be associated with opposite effects. Our unexpected results reveal a potential disturbing role of emotion in immediate situations. Since eye movements are considered as a reliable measure of selective attention, and particularly for measuring selective attention to emotional stimuli (Isaacowitz et al., 2006; Sears et al., 2019) including eye-tracking measures in further works would help determine the extent to which attentional factors are involved in the trade-offs we observed for focal and diffuse emotional pictures. Additional studies are needed to assess whether such effects are also present in longer delay situations.

Finally, since we used negative pictures, related to withdrawal behaviors, it could also be interesting to reproduce our experiments with positive emotions (e.g., joy) but also with negative emotions associated with approach behaviors (e.g., anger).

## Data availability statement

The raw data supporting the conclusions of this article will be made available by the authors, without undue reservation.

## Ethics statement

The studies involving human participants were reviewed and approved by the Ethics Committee of the University Savoie Mont Blanc. The patients/participants provided their written informed consent to participate in this study.

## Author contributions

DB, PH, and JG contributed to the study design and methodology. DB and MS-M performed investigation, testing, data collection, and interpretation under the supervision of PH and JG. DB and JG drafted the manuscript. PH provided critical revisions. All authors contributed to the article and approved the submitted version.

## Acknowledgment

We thank Jessica Bourgin for her careful review of the final manuscript.

## Footnotes

1
https://www.qualtrics.com/fr/

2
https://www.prolific.co/

3
https://www.adobe.com/fr/products/photoshop.html

4
https://lab.js.org/

5
https://open-lab.online/
